# Biological activities of a recombinant fortilin from *Fenneropenaeus merguiensis*

**DOI:** 10.1371/journal.pone.0239672

**Published:** 2020-10-01

**Authors:** Ureporn Kedjarune-Leggat, Uraipan Saetan, Anchana Khongsaengkaeo, Sudarat Suwannarat, Panchalika Deachamag, Monwadee Wonglapsuwan, Rawiwan Pornprasit, Wanwisa Thongkamwitoon, Parujee Phumklai, Jirapan Chaichanan, Wilaiwan Chotigeat

**Affiliations:** 1 Department of Oral Biology, Faculty of Dentistry, Prince of Songkla University, Hatyai, Songkhla, Thailand; 2 Department of Molecular Biotechnology and Bioinformatics, Faculty of Science, Prince of Songkla University, Hatyai, Songkhla, Thailand; 3 Mahidol University-Bio Innovation Building, Mahidol University, Nakhon Pathom, Thailand; 4 Center for Genomics and Bioinformatics Research, Faculty of Science, Prince of Songkla University, Hatyai, Songkhla, Thailand; MAHSA University, MALAYSIA

## Abstract

Human Fortilin, an antiapoptotic protein, has also been implicated in several diseases; however, several potential uses of fortilin have also been proposed. Bearing the implications of fortilin in mind, fortilin analog, which has no complication with diseases, is required. Since a recombinant full-length fortilin from *Fenneropenaeus merguiensis* (*rFm-Fortilin* (FL)) reported only 44% (3e^-27^) homologous to human fortilin, therefore the biological activities of the Fm-Fortilin (FL) and its fragments (F2, F12, and F23) were investigated for potential use against HEMA toxicity from filling cement to pulp cell. The rFm-Fortilin FL, F2, 12, and F23 were expressed and assayed for proliferation activity. The rFm-Fortilin (FL) showed proliferation activity on human dental pulp cells (HDPCs) and protected the cells from 2-hydroxy-ethyl methacrylate (HEMA) at 1–20 ng/ml. In contrast, none of the rFm-Fortilin fragments promoted HDPC growth that may be due to a lack of three conserved amino acid residues together for binding with the surface of Rab GTPase for proliferative activity. In addition, rFm-Fortilin (FL) activated mineralization and trend to suppressed production of proinflammatory cytokines, including histamine (at 10 ng/ml) and TNF-α (at 100 ng/ml). Besides, the rFm-Fortilin (FL) did not mutate the Chinese hamster ovary (CHO) cell. Therefore, the rFm-Fortilin (FL) has the potential use as a supplementary medical material to promote cell proliferation in patients suffering severe tooth decay and other conditions.

## Introduction

Translationally controlled tumor protein (TCTP) is an antiapoptotic protein, also known as fortilin, which has 172 amino acids and its sequence is significantly conserved among eukaryotes; the majority of fortilin is found in the nucleus of the cell [1, 2]. Although fortilin does not have a structure similar to inhibitors of apoptosis proteins or the Bcl2 family, fortilin has been proposed to control cell apoptosis [2]. In addition, fortilin was highly expressed as an antiapoptotic protein in several human cancers, including cutaneous squamous cell carcinoma (SCC) [3], ovarian cancer [4], and gliomas. Since A431 cells present low expression of fortilin, they also show decreased cell proliferation. Therefore, the fortilin gene was suggested as a target in skin SCC therapy [3]. In another study, fortilin exhibited cell growth promotion, calcium (Ca^2+^) binding and anti-pathogenic activities, facilitated atherosclerosis development and contributed to arterial hypertension [5].

Although fortilin has been implicated in several diseases, diverse potentially positive effects have also been proposed. For example, fortilin was found to inhibit migration and apoptosis of vascular smooth muscle cells. Consequently, it was introduced as a crucial vaso-protective protein with therapeutic effects [6]. In addition, fortilin was reported to increase the activity of peroxiredoxin-1 (PRX1) by protecting the enzyme from phosphorylation and it was also found to prevent liver destruction induced by alcohol in transgenic animals; accordingly, it was suggested to have potential benefits for humans who have acute liver damage induced by alcohol [7]. In animal, fortilin also found to protect shrimp from White Spot Syndrome [8].

In dentistry, HEMA is a monomer in resin-based restorative materials that is released during the restoration of dental cavities [9]. A recombinant Glutathione-S-transferase-Fortilin (GST-Fortilin) from *Fenneropenaeus merguiensis* (Fm-GST-Fortilin) reduced the toxicity to dental pulp cells that caused by 2-Hydroxy-ethyl methacrylate (HEMA) were reported [9]. Fm-GST-Fortilin was also reported to promote osteoblast cell proliferation and differentiation function [10]. Besides, human fortilin activates histamine-releasing via IgE based [11], and the fortilin was reported related to human cancer. Therefore, it is essential to investigate the properties of rFm-Fortilin, whether the rFm-Fortilin activates histamine-releasing, trigger genome mutation, and cytoprotective mechanism for further potential applications.

The potential features of rFm-Fortilin for application as a supplement in dental or medical materials were assessed in this study. Thus, the full-length cDNA of *rFm-Fortilin* (FL) (without GST) was generated from the ovarian tissue of *F*. *merguiensis* [12]. In addition, the rFm-Fortilin fragments were designed by an alignment of the rFm-Fortilin (FL) with the human fortilin or Human histamine-releasing factor (HRF). The conserved regions were selected and divided the rFm-Fortilin (FL) into three fragments, namely rFm-Fortilin (F12), rFm-Fortilin (F2), and rFm-Fortilin (F23) to find the proliferative region, histamine-releasing region, or cancer-related region that may contain. The role of rFm-Fortilin (FL) and its fragments were evaluated for pulp cell proliferation and cytotoxic protection. We select the later method because it can represent the clinical application of this protein when we added this protein in dental material that already has HEMA. Then, the recombinant protein, which has the best activities, was further studied for calcium deposition, histamine-releasing, TNF-alpha inhibition (a cytoprotective mechanism), and genome mutation.

## Materials and methods

### Production and purification of the rFm-Fortilin (FL)

We constructed a recombinant plasmid containing the nucleotides encoding *Fm-Fortilin*. The *rFm-Fortilin* nucleotide sequence from Loongyai and colleagues [12] was synthesized and cloned in pET29a+ at the *Nde*I and *Kpn*I sites and transformed in *Escherichia coli* BL21(DE3). To prepare a seed culture for *rFm-Fortilin* expression, glycerol stock of *E*. *coli* BL21(DE3) harboring pET29a-Fm-Fortilin was inoculated into 2 ml LB medium (1% (w/v) Tryptone, 0.5% (w/v) yeast extract, and 1% (w/v) NaCl supplemented with 30 μg/ml kanamycin. The culture was grown at 37°C with shaking at 180 rpm for 16–18 h and transferred to 50 ml of LB medium in a shake flask. Cultivation was performed at 37°C with shaking at 180 rpm until the absorbance at 600 nm was 0.5–0.6. Isopropyl β-D-thiogalactopyranoside (IPTG) was added to a final concentration of 0.4 mM in the culture, which was further incubated at 18°C for 24 h. The bacterial cells were harvested by spinning at 4,000 × g for 20 min at 4°C. The cell pellet was solubilized in 5 ml of lysis buffer (50 mM Na_2_PO_4_ pH 7.6, 10 mM Tris-HCl pH 7.6, 1 mg/ml lysozyme), incubated at 4°C for 30 min and sonicated (20×10s, 200 to 300 W). The lysed cells were centrifuged at 12,000×g for 20 min at 4°C, and the supernatant was harvested and filtered through a 0.2 μm membrane.

One milliliter HiTrap DEAE FF column (GE Healthcare, Thailand)was used to purify the rFm-Fortilin (FL), and the column was equilibrated with 10 column volumes of 20 mM Tris-HCL at pH 7.6. The filtered supernatant was loaded into the column at 1 ml/min by an AKTA Start (GE Healthcare) and washed with wash buffer (20 mM Tris-HCL, 0.1 M NaCl at pH 7.6) for 10 column volumes. The protein was eluted with 10 column volumes of 20 mM Tris-HCL, pH 7.6, containing 0.5 M NaCl at pH 7.6, and 1 ml fractions were collected. The purity of the protein was checked by 15% SDS-PAGE gel, and the purified protein was pooled and kept at -80°C for further study.

### Production and purification of the rFm-Fortilin (F12, rFm-Fortilin (F2) and rFm-Fortilin (F23)

**Fig 1:**
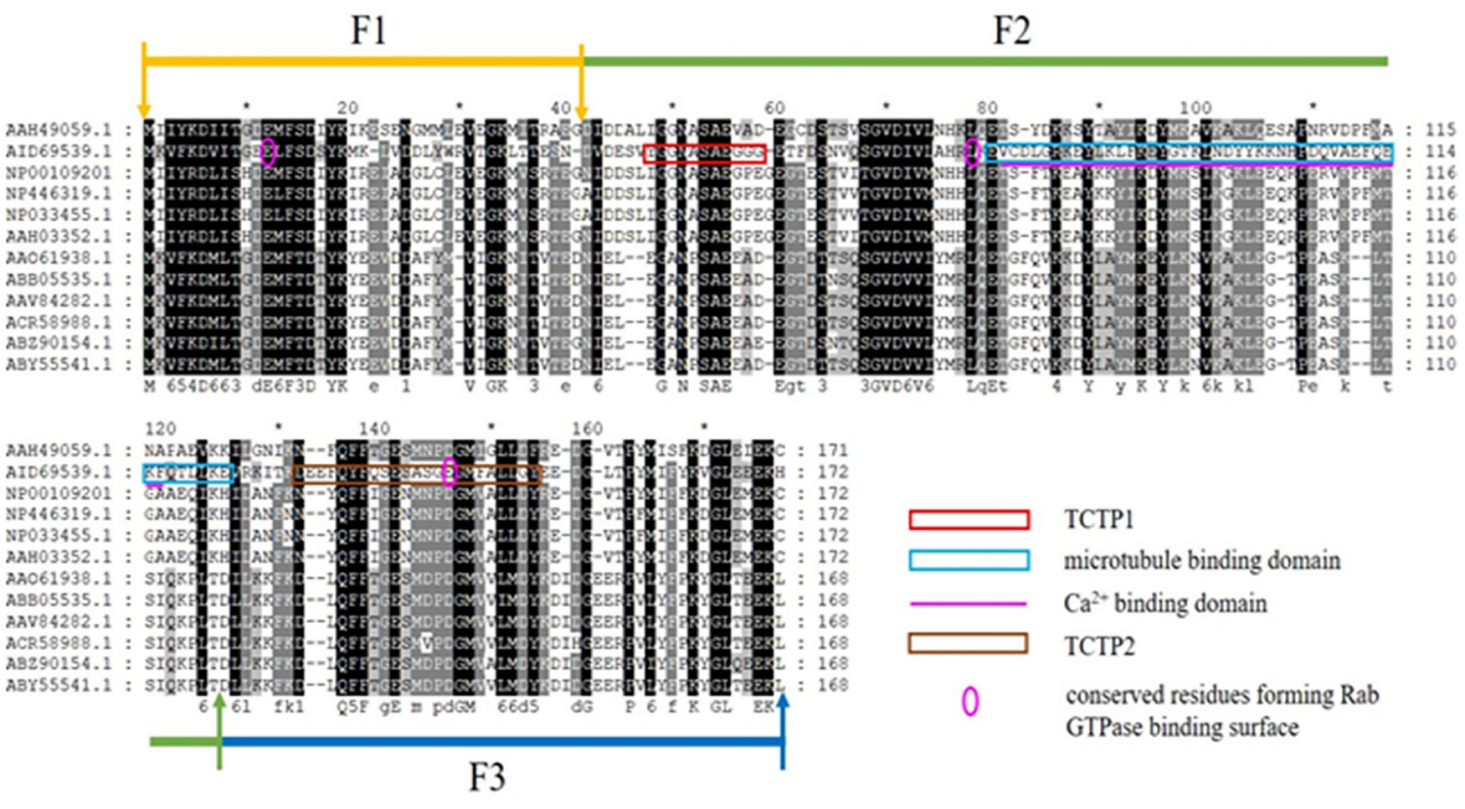
Multiple alignments of TCTP protein among different species. The conserved residues are highlighted in black shadow. Fm-Fortilin fragment12 shows in yellow and green, Fm-Fortilin fragment2 is green and Fm-Fortilin fragment 23 is green and blue. The following species were aligned as *Mus musculus* (NP_033455.1), *Danio rerio* (AAH49059.1), *Penaeus japonicus* (ABZ90154.1), *Homo sapiens* (AAH03352.1), *Rattus norvegicus* (NP_446319.1), *Pan troglodytes* (NP_001092016.1), *Penaeus vannamei* (ABY55541.1), *Penaeus indicus* (ACR58988.1), *Penaeus monodon* (AAO61938.1), *Penaeus merguiensis* (AAV84282.1), *Penaeus chinensis* (ABB05535.1).

The *Fm-Fortilin* (FL) gene was divided into three fragments (F12, F2 and F23) according to the regions mainly conserved in fortilin genes of other species (Fig 1). All fragments were amplified from *rFm-Fortilin* (FL) with specific primers that contained the restriction sites *Nde*I and *Xho*I. After digestion and purification, all fragments were subsequently cloned into the pET29a+ vector (GenScript) and further verified by sequencing. The *rFm-Fortilin* (F12, F2, and F23) plasmids were transformed in *E*. *coli* BL21(DE3).

To express the *rFm-Fortilin* F12, F2 and F23 fragments, the *E*. *coli* BL21(DE3) containing pET29a-*Fm-Fortilin* was grown overnight at 37°C in 30 ml of LB broth containing 30 μg/ml kanamycin for 16–18 h with shaking at 180 rpm. The starter cultures were transferred to 300 ml of LB broth and incubated until the cells reached an OD.600 of 0.5–0.6. The IPTG was added until a final concentration of 1 mM in the culture. Cells were incubated at 30°C for an additional 16–18 h. Bacterial cells were harvested by centrifugation at 4,000×g for 20 min and sonicated (20×10 s, 200 to 300 W) in lysis buffer (50 mM NaH_2_PO_4_, pH 8.0, 300 mM NaCl, and 5 mM imidazole). The lysed cells were centrifuged at 12,000×g for 20 min at 4°C, and the supernatants were separated and examined on 15% SDS-PAGE gel using Coomassie brilliant blue staining.

The supernatants were filtered with a 0.2 μm membrane and loaded into a 1 ml HisTrap FF column (GE Healthcare) at 1 ml/min by an AKTA Start (GE Healthcare) to purify the rFm-Fortilin F12, F2 and F23 fragments. The 1 mL HisTrap column was equilibrated and washed with 50 column volumes of wash buffer (20 mM NaH_2_PO_4_, 0.5 M NaCl, 20 mM Imidazole, pH 7.4). The column was eluted with 10 column volumes of elution buffer (20 mM NaH_2_PO_4_, 0.5 M NaCl, 500 mM imidazole, pH 7.4), and 1 mL of each fraction was collected. The purity of the protein in the fractions was checked on 15% SDS-PAGE and kept at -80°C.

### Cultures of HDPCs

Normal human third molars were picked up from one subject at the Dental Hospital, Faculty of Dentistry, Prince of Songkla University, with consent forms approved by the Research Ethics Committee (Reference No. EC5505-15-L), Faculty of Dentistry, Prince of Songkla University. Primary pulp cells were prepared using enzymatic digestion [9]. Briefly, the pulp tissue was minced and digested for 1 h at 37°C in a solution containing 3 mg/ml of collagenase type I and 4 mg/ml of dispase. Then, the cells were separated by centrifugation, cultured in alpha-modified Eagle’s medium (α-MEM) supplemented with 20% fetal calf serum (FCS), 100 μM L-glutamate, 100 μg/ml streptomycin, 100 units/ml penicillin, and 100 μM L-ascorbic acid 2-phosphate (Sigma-Aldrich, St Louis, MO, USA), and incubated at 37°C with 5% CO_2_. All experiments in this work were carried out using human dental pulp cells (HDPCs) taken from passages three to six.

### Effect of rFm-Fortilin on HDPCs viability

The effect of rFm-Fortilin on pulp cell viability was determined by the 3-(4,5-dimethylthiazol-2-yl)-2,5-diphenyltetrazolium bromide (MTT) assay. Cells were cultured on 96-well plates at a density of 1 × 10^4^ cells/well in a humidified atmosphere of 5% CO_2_ at 37°C. After 24 h, HDPCs were grown in culture medium supplemented with rFm-Fortilin (FL), rFm-Fortilin (F2), rFm-Fortilin (F12) and rFm-Fortilin (F23) ranging from 1 ng/ml- 20 μg/ml and control cells in medium only for 24 h and 72 h. After 24 h and 72 h, the media in each well was changed with 200 μl of fresh medium containing 10 mM HEPES (pH 7.4) and 50 μl MTT solution (5 mg/ml in PBS) and incubated for 3 h at 37°C in darkness. The solution after incubation was removed, and then 200 μl of DMSO and 25 μl of Sorensen’s glycine buffer (0.1 M glycine plus 0.1 M NaCl equilibrated to pH 10.5 with 0.1 M NaOH) were added. Formazan production was measured based on the absorption at 570 nm. The OD values of medium were corrected for a blank (medium only) of the experimental groups, were divided by the control (cells cultured with medium only) and expressed as a percentage of the control, which presented the percentage of viable cells.

### Cytoprotective effect of rFm-Fortilin on HEMA-treated HDPCs

Cells at a density of 1 × 10^4^ cells/well were cultured in the normal culture medium on 96-well plates in a humidified atmosphere of 5% CO_2_ at 37°C. After 24 h, the medium was replaced with a medium mixed with a combination of 8 mM HEMA and rFm-Fortilin (FL), rFm-Fortilin (F2), rFm-Fortilin (F12) and rFm-Fortilin (F23) ranging from 1 ng/ml-20 μg/ml and incubated for 48 h. The positive control used 8 mM HEMA alone. After that, the normal fresh media was replaced. The cytoprotective effect of the rFm-Fortilin (FL), rFm-Fortilin (F2), rFm-Fortilin (F12) and rFm-Fortilin (F23) was determined by an MTT assay as described above.

### Determination of mineralization promotion

Calcium deposition was modified from Bakopoulou and team [13] and determined by the alizarin red staining (ARS) assay (Sigma, Life Science, USA). Three experimental groups were utilized, including the control, which consisted of pulp cells in the inductive medium; the positive group, which consisted of pulp cells treated with 8 mM HEMA in the inductive medium for 24 h; and the tested group, which consisted of pulp cells treated with 8 mM HEMA and 19.2 ng/ml of rFm-Fortilin (FL) in the inductive medium for 24 h. The inductive medium was composed of 10 mM β-glycerophosphate, 100 units/ml penicillin, 100 mg/ml streptomycin and 100 mg/ml antibiotic-antimycotic in α-MEM with 20% FCS. Pulp cells were seeded at 3×10^3^ cells/well in a 6-well plate and fed with 2 ml/well of the inductive medium at 37°C in 5% CO_2_ and a humidified atmosphere. After the first 24 h, the medium was replaced with the inductive medium containing the specific components according to the designed group as previously described above and then changed every 2 days. After 7, 14, 21 and 28 days, the medium was removed and the cells were washed with PBS at pH 7.4, fixed in 10% (v/v) formaldehyde (Sigma, Life Science, USA) at room temperature for 15 min and washed twice with 1 ml/well of 40 mM ARS at pH 4.1 in ultrapure water at room temperature for 30 min. After the removal of ARS, the plate was washed in ultrapure water with shaking for 5 min, and the washing was repeated four times. Excess water was removed, and the plate was stored at -20°C before dye extraction. Staining was evaluated by adding 100 μl of 10% (v/v) acetic acid to each well and incubated at room temperature for 30 min with shaking. The cell mixture of each well was then transferred to a 1.5 ml tube with 100 μl mineral oil (Sigma, Life Science, USA), heated at 85°C for 10 min and placed on ice for 5 min. The slurry was centrifuged at 20,000 × g for 15 min, and then 100 μl of the supernatant was transferred to each well of a 96-well plate and added with 40 μl of 10% (v/v) ammonium hydroxide. The sample was measured by an ELISA reader at 405 nm against its own blank.

### Effect of rFm-Fortilin (FL) on histamine and TNF-α release

Macrophage cell lines (NR8383, ATCC) were cultured in F-12K medium supplemented with 15% FBS and 1% penicillin-streptomycin at 37°C in the presence of 5% CO_2_. To determine the amount of secreted histamine and TNF-α, macrophage cells were determined the amount of secreted histamine and TNF-α plated at a density of 3×10^4^ cells/well in a 96-well cell culture plate and pretreated with LPS (0.5 μg/ml) for 1 h. Various concentrations of rFm-Fortilin (FL) protein at 0.001, 0.01, 0.1, 1, 10 and 20 μg/ml were added to the cells pretreated with LPS and incubated for 48 h. The rFm-Fortilin (FL) was added to untreated cells as the negative control, and cells treated with LPS alone were served as a positive control. After 48 h of treatment, cell culture supernatants were collected and stored at -80°C for further cytokine analysis. The concentrations of histamine and TNF-α were quantified using an ELISA kit (Histamine Assay kit, colorimetric) and a TNF-alpha Human Simple Step ELISA kit (Abcam, UK) according to the manufacturer's instructions.

### Determination of rFm-Fortilin genotoxicity

CHO cells were seeded at 1x10^5^ cells/well in a 48-well plate and incubated for 36–40 h. The cells were exposed to a concentration of either 0.5 or 1 μg/ml of rFm-Fortilin (FL) protein for 24, 48, or 72 h at 37°C, after which the medium containing the rFm-Fortilin (FL) protein was removed. 1-methyl-3-nitro-1-nitrosoguanidine (MNNG) (Tokyo Chemical Industry Co., Ltd, Japan) and 7,12-dimethylbenz (a) anthracene (DMBA) (Sigma Aldrich, US) mutagens were used as positive controls, while PBS and DMSO were used as negative controls. The cells were cultured in DMEM for 8 days. Mutant cells were identified by treatment with 5 μg/ml 6-thioguanine (6-TG) (Sigma Aldrich, US) in DMEM. After 10 days of growth, the cells were stained with Trypan blue and counted to determine the mutant frequency.

### Statistical analysis

All data were expressed as the means ± standard deviation (SD). One-way ANOVA and Tukey’s multiple comparisons were used to investigate the differences among the experimental groups.

## Results

### Production and purification of the rFm-Fortilin in *E*. *coli* BL21(DE3)

**Fig 2:**
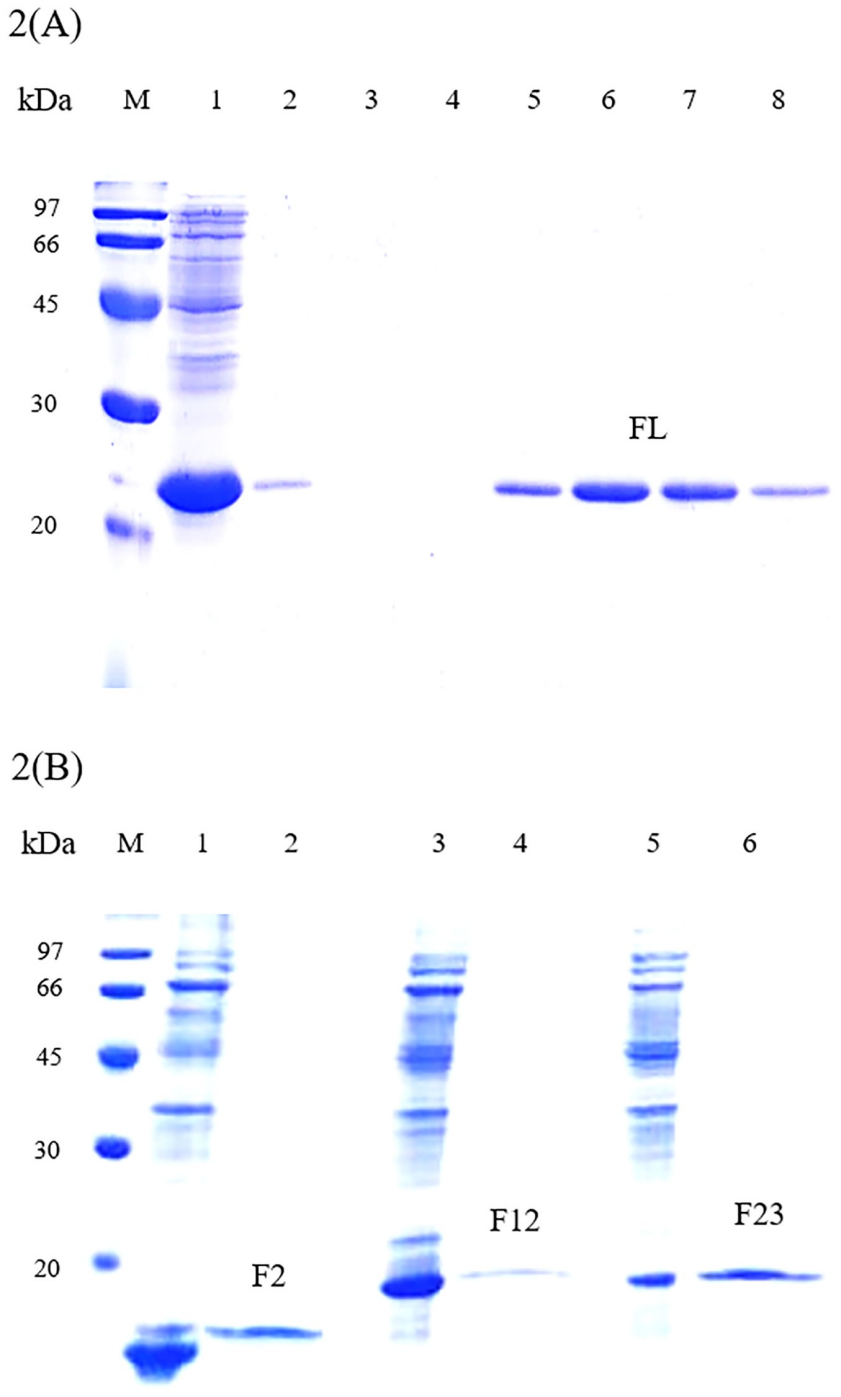
rFm-Fortilin and its fragments separated on 15% SDS-PAGE. **(A)** rFm-Fortilin (FL) transformed in *E*. *coli* BL21(DE3) harboring pET29a-Fortilin-Full Length was analyzed on 15% SDS-PAGE and stained with Coomassie brilliant blue. Lane M: Molecular weight marker; Lane 1: Soluble fraction of crude extract; Lane 2–4: Eluted fractions from DEAE column; Lane 5–8: Purified rFm-Fortilin from HiTrap DEAE FF column. **(B)** rFm-Fortilin (F2), rFm-Fortilin (F12), rFm-Fortilin (F23) transformed in *E*. *coli* BL21(DE3) harboring pET29a-Fortilin (F2), Fortilin (F12), and Fortilin (F23) were analyzed on 15% SDS-PAGE. Lane M: Molecular weight marker; Lane 1: crude rFm-Fortilin(FL); Lane 2: purified rFm-Fortilin(F2); Lane 3: crude rFm-Fortilin (F12); Lane 4: purified rFm-Fortilin (F12); Lane 5: crude rFm-Fortilin (F23); and Lane 6: purified rFm-Fortilin (F23).

The *rFm-Fortilin* (FL) gene and its fragments; *rFm-Fortilin* (F12), *rFm-Fortilin* (F2), and *rFm-Fortilin* (F23) cloned in pET29a+ are represented in Fig 1. An in silico analysis determined that the molecular weights of rFm-Fortilin (FL), rFm-Fortilin (F2), rFm-Fortilin (F12), and rFm-Fortilin (F23) were approximately 19.2, 8.5, 13.2, and 14.5 kDa, respectively. Purification of rFm-Fortilin (FL) with the HiTrap^™^ DEAE FF column effectively bound rFm-Fortilin (FL) protein to the DEAE resin, thus capturing negatively charged protein molecules [14]. The increased purity of the target rFm-Fortilin (FL) protein is shown in Fig 2A and showed higher molecular weight on SDS- PAGE than the computer prediction as previously reported [12]. The successful purification of rFm-Fortilin (F12), rFm-Fortilin (F2) and rFm-Fortilin (F23) with the 1 ml HisTrap FF column are shown in Fig 2B.

### Effect of rFm-Fortilin (FL, F11, F2, and F23) on HDPC viability

**Fig 3:**
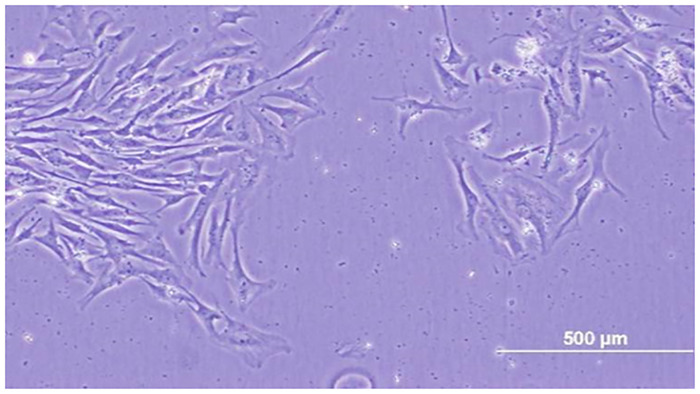
Primary HDPCs. HDPCs were cultured for 5 days and investigated with a phase-contrast microscope at 100x magnification.

The isolated HDPCs were cultured under 5% CO_2_ for 5 days at 37°C. The cells attached to the surface of the cell culture plate and formed the colony (Fig 3).

**Fig 4:**
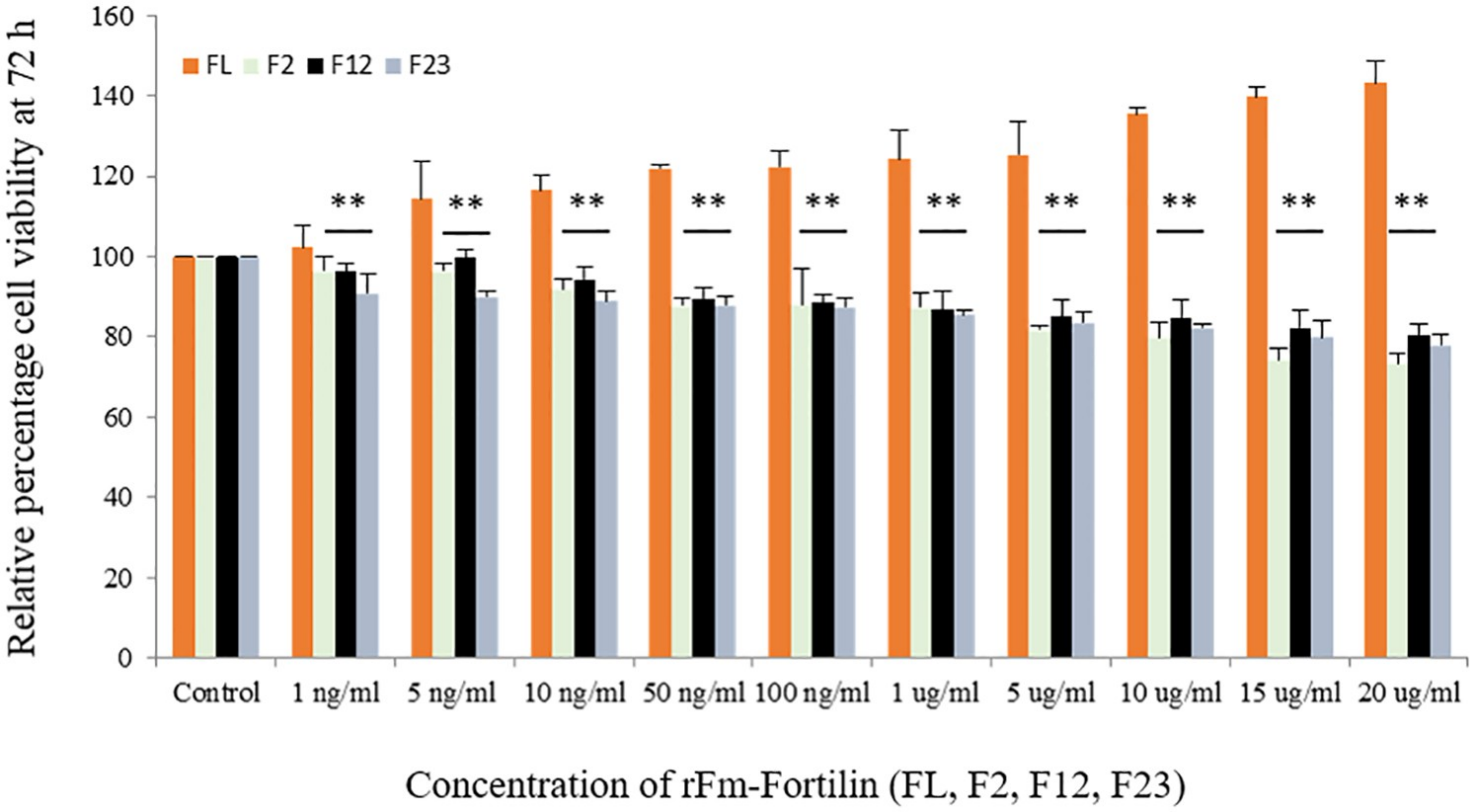
Effect of rFm-Fortilin (FL, F2, F12, and F23) on HDPC viability. Cells were treated for 72 h with rFm-Fortilin (FL, F2, F12, and F23) ranging from 1 ng/ml to 20 μg/ml. An MTT assay was used to evaluate the cell viability. The experiments were repeated six times, and the results represented the values of the means ± SD. *p < 0.05, **p < 0.01.

Results in Fig 4 revealed that rFm-Fortilin (FL) ranging from 1 ng/ml to20 μg/ml exhibited no cytotoxicity to HDPCs after exposure for 24 and 72 h. On the other hand, it could promote cell proliferation due to the percentage of viable cells was higher than one hundred percent, when compared with the control group. Cells treated with rFm-Fortilin (FL) at both 24 and 72 h showed the same pattern of increasing percentages of viable cells with increased rFm-Fortilin (FL) concentrations. The viability of cells treated with rFm-Fortilin (FL) ranging from 1 ng/ml to 20 μg/ml for 72 h was significantly (*p* < 0.05) higher than that of the other groups. To design the new fragment of the rFm-Fortilin (FL), which contains proliferative activity and no histamine-release, which may occur. So the cDNA was divided into three pieces (F12, F2, F23) according to the conserved region of human TCTP showed in Fig 1. However, rFm-Fortilin fragments F12, F2, and F23 did not have proliferative activity. The proliferative activity of the rFm-Fortilin (FL) may require the proper folding to bind a substrate while the protein fragments possibly too small to form a good structure for activities.

### Effect of rFm-Fortilin (FL), rFm-Fortilin (F2), rFm-Fortilin (F12) and rFm-Fortilin (F23) on the cytotoxicity of HEMA in HDPCs

**Fig 5:**
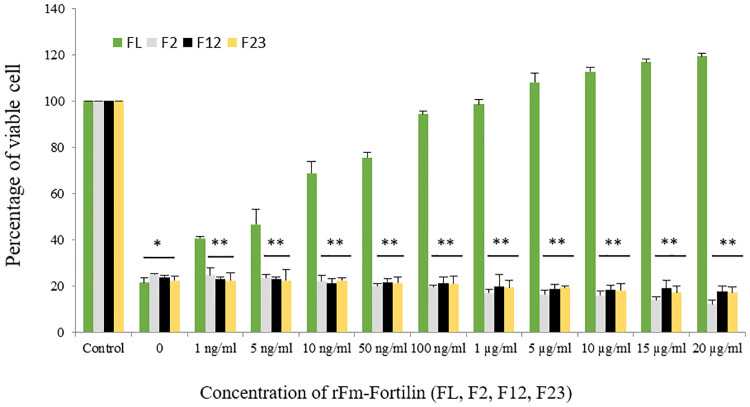
Cytoprotective effect of rFm-Fortilin (FL, F1, F12, and F23) on HEMA-treated cells. Cells were cultured in medium containing 8 mM HEMA and rFm-Fortilin (FL, F1, F12, and F23) at concentrations from 1 ng/ml to 20 μg/ml. MTT assay was used to evaluate cell viability. The experiments were performed six times, and the results are shown as the means ± SD (*p < 0.05, **p < 0.01).

The protective effect of rFm-Fortilin (FL) against HEMA toxicity was described as the percentage of viable cells (Fig 5). The cell viability in the groups treated with HEMA and rFm-Fortilin (FL) was significantly higher than in other groups treated with HEMA only. Viability percentages were significantly higher (*p* < 0.05) than among cells treated with rFm-Fortilin (FL) at concentrations from 1 ng/ml to 20 μg/ml compared with the cells treated with HEMA only. Pulp cells treated with HEMA and rFm-Fortilin (FL) from 100 ng/ml to 20 μg/ml had no significant differences in percentage viability when compared to untreated cells. However, the rFm-Fortilin (F2), rFm-Fortilin (F12) and rFm-Fortilin (F23) concentrations ranging from 1 ng/ml to 20 μg/ml did not protect cells from HEMA toxicity, the effect on cell death increased at higher concentrations. Since the cytoprotective activity and proliferative activity were the major requirements of this study, therefore the rFm-Fortilin (F2), rFm-Fortilin (F12) and rFm-Fortilin (F23) were not further evaluated for other biological properties.

### Mineralization of rFm-Fortilin (FL)

**Fig 6:**
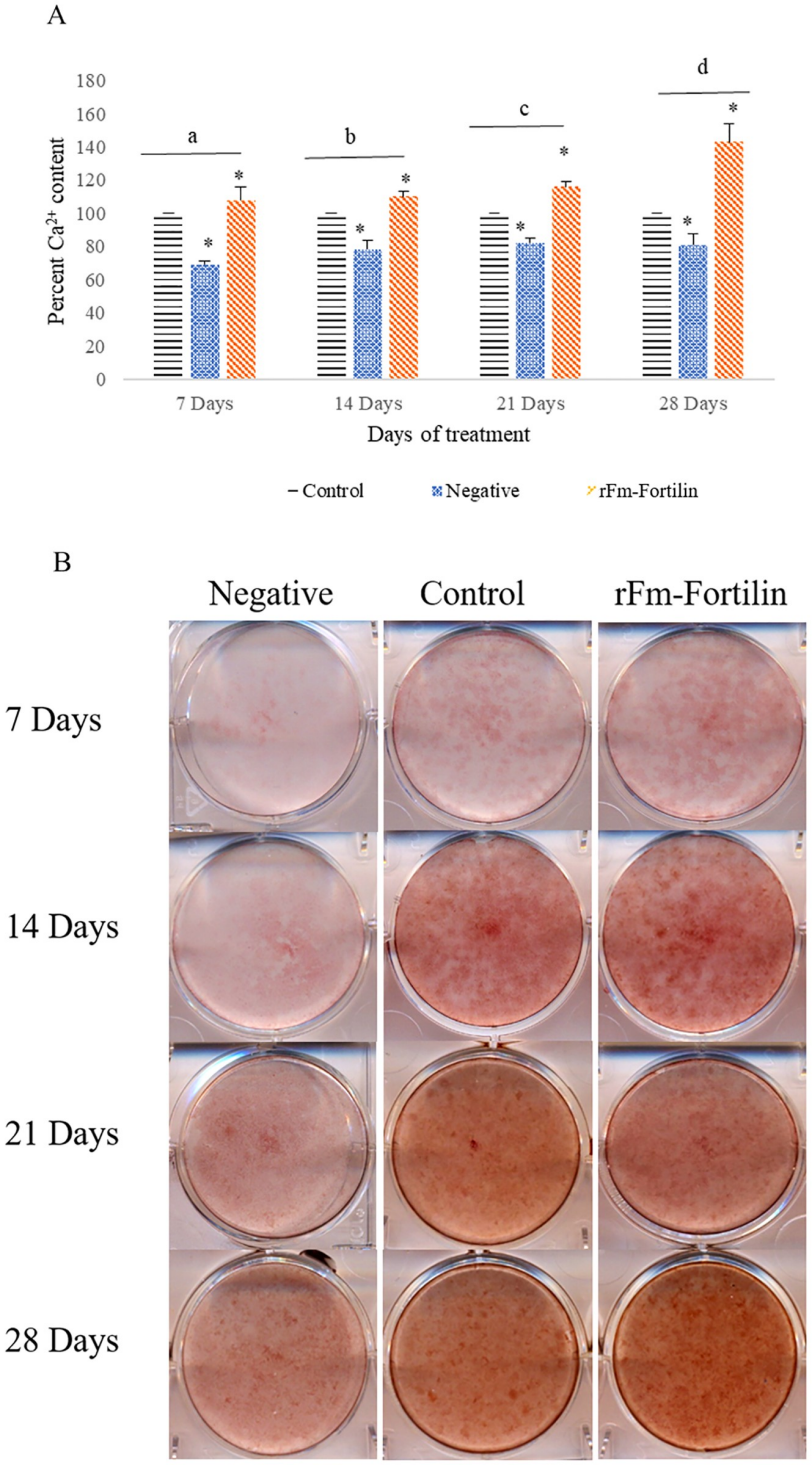
Mineralization of rFm-Fortilin. (A) Percentage of Ca^2+^ content, (B) Precipitate of calcium in the medium of pulp cells culture. The control consisted of pulp cells in the inductive medium, the negative group consisted of cells treated with 8 mM HEMA for 24 h in inductive medium, and the treatment group consisted of cells treated with 8 mM HEMA for 24 h in inductive medium, after which rFm-Fortilin (FL) was added.

The rFm-Fortilin (FL) was added to the cells treated with 8 mM HEMA for 24 h in the inductive medium, and the calcium content was analyzed. The calcium content was significantly higher in the presence of rFm-Fortilin (FL) at 7 days and exponentially increased after 28 days (Fig 6A), and calcium precipitation was shown in Fig 6B.

### Histamine and TNF-α releasing assay of rFm-Fortilin (FL)

**Fig 7:**
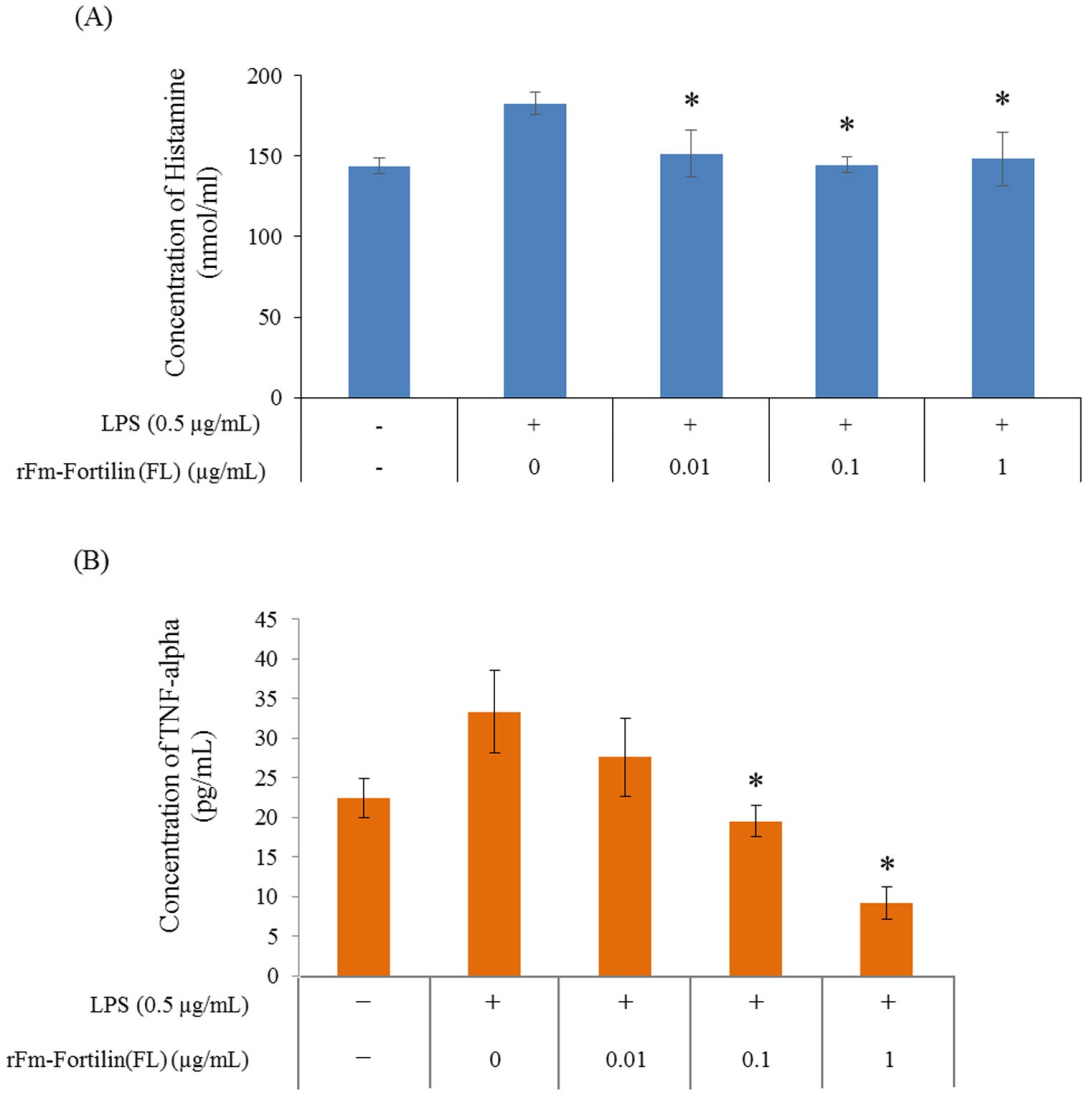
rFm-Fortilin suppressed the induction of proinflammatory cytokines by LPS activated macrophages. Histamine (A) and TNF-α (B) were determined with an enzyme-linked immunosorbent assay (ELISA). The various concentrations of rFm-Fortilin (0.01, 0.1, and 1 μg/ml) were assayed for inhibition of the histamine-releasing and TNF-α. The presented data are the mean ± SD of triplicate experiments (*p<0.05).

Suppression of histamine and TNF-α release from macrophage cells by rFm-Fortilin (FL) was determined after stimulation with LPS. Inhibition of the histamine-releasing occurred in a dose-dependent manner from 10 ng/ml to 1 μg/ml (Fig 7A). TNF-α, a proinflammatory cytokine, was significantly inhibited by rFm-Fortilin (FL) from 10 ng/ml to 1 μg/ml (Fig 7B).

### Genotoxicity of the rFm-Fortilin (FL)

**Fig 8:**
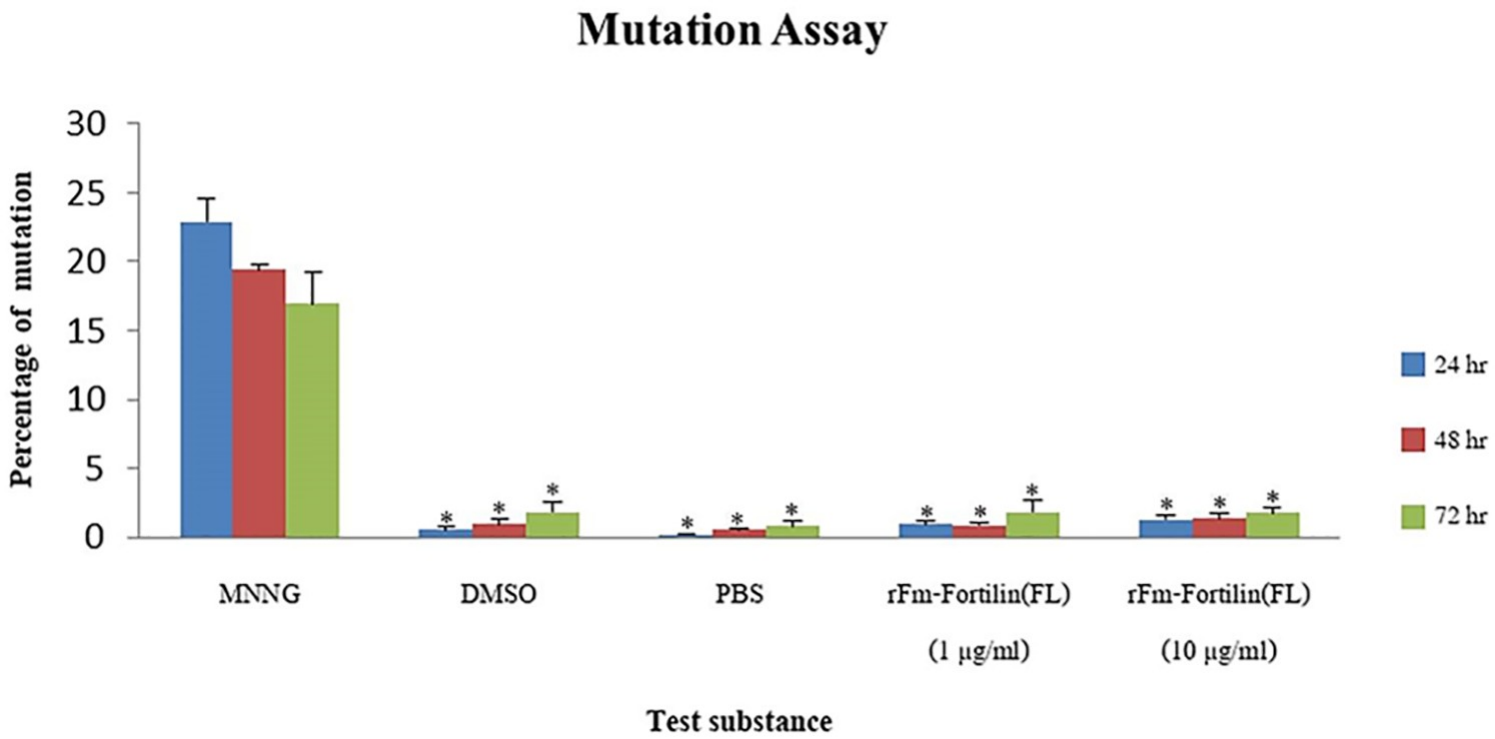
Mutation frequency of CHO cells after treatment with MNNG, DMSO and rFm-Fortilin. CHO cells were activated with MNNG (positive control), DMSO (negative control), PBS (negative control), 1 and 10 μg/ml of rFm-Fortilin (FL). The mutation frequency was calculated. *Denotes a significant difference of test substance compared to MNNG using the t-test (*p*<0.005).

CHO cells were treated with a mutagen, MNNG. The percentage of mutation was approximately 23% at 24 h and decreased as the incubation time increased. No significant difference in the percentage mutation among rFm-Fortilin (FL)-treated cells were found when compared with the negative controls DMSO and PBS, shown in Fig 8.

## Discussion

TCTP, also named fortilin, has been found in various organisms, including mice, humans, yeast, and parasites [2, 15–20]. Fortilin has been reported [15] to have various functions or mechanisms, such as anti-apoptosis [2, 21], phosphorylation regulation [22], tumor transformation or proliferation [23–25], and oxidative stress reduction [26]. The results of this study confirmed that rFm-Fortilin could promote HDPC proliferation, which is consistent with the rFm-glutathione S-transferase (GST)-TCTP in previous studies [9].

The fragments (F12, F2, F23) of rFm-Fortilin were produced; however, no proliferative activity was found. The conserved amino acid residues E12, L75, and E143 of the *Stm*TCTP (*Stichopus monotuberculatus* TCTP) were required to bind with the surface of Rab GTPase for proliferative activity [27]. The fragment 12 contained the residue E12, L75. The residue L75 was in F2 while the fragment 23 contained residue L75 and D146. E143 was reported in sea cucumber while D146 was in *Penaeus* sp. (Fig 1). Only the rFm-Fortilin (FL), which contained all 3 conserved residues, had proliferative activity. Taken together indicated that three binding sites E12, L75, E146 were required for proliferative activity.

Other studies also reported that cell growth and proliferation required TCTP. For example, the in vitro and in vivo experiments found that TCTP activated the binding of TCF-4 and β-catenin and then increased glioma cell growth [28]. *Drosophila* TCTP (*d*TCTP) was reported to control the activity of the Ras homolog enriched in brain (Rheb) pathway, which is the growth regulator of Tuberous sclerosis (TSC) [29]. TCTP and P23 from mouse fibroblasts have been reported to be associated with microtubules in the G1, S, G2, and M-phases of the cell and are also temporally bound to the spindle at the M-phase [30]. Besides, polo-like kinase Plk phosphorylated TCTP, which caused the destabilization of the microtubules and movement of the spindle during metaphase to anaphase. Furthermore, TCTP was mutated to a non-phosphorylation structure, which caused an incomplete cell cycle during mitosis [22].

HEMA is a component in dental resin composite material, and it has been reported to be released in the mM range from resin composite into the pulp space after polymerization within 24 h [31–33]. The in vitro toxicity of HEMA has been reported in several studies. For example, HEMA was cytotoxic to the cell because of the production of ROS and oxidation of double-strand DNA breaks [32, 34]. The creation of ROS can cause inflammation and apoptosis, while the ROS were crucial for activating the kappa B (NF-kB) factor, thus providing a protective role in apoptosis [35]. Besides, HEMA caused ROS production, while glutathione was reduced [36]. Another study supporting HEMA-induced apoptosis suggested that the dose of ROS was related to fragmentation of DNA and induction of micronuclei [37].

The ability of HEMA to induce apoptosis in vitro after 24 h was also reported [34]. The results of this study confirmed that HEMA at 8 mM was cytotoxic to pulp cells. This finding is consistent with the report of Wanachottrakul *et al*. [9], who showed that HDPCs exposed to HEMA (at 0–16 mM) exhibited a dose-dependent increase in apoptotic cells.

In addition to its proliferative activity, rFm-Fortilin (FL) showed cytoprotective activity against HEMA. In this study, HEMA-exposed pulp cells treated with 1 ng/ml rFm-Fortilin (FL) showed comparable viability with unexposed cells. This concentration was the lowest at which rFm-Fortilin (FL) effectively protected cells against 8 mM HEMA, and this finding is correlated with previous research [9]. Since ROS can oxidize cell components, including oxidized DNA results in DNA fragmentation and base modification, oxidized proteins result in non-function or form toxic aggregation with lipid, which leads to several diseases and cell death [38]. Accumulation of ROS in mitochondria cause loss of mitochondrial cell membrane permeability, result in cell swelling, the release of cytochrome C and eventually lead to autophagy, apoptosis or necrosis [39]. ROS is induced to autophagy by several components such as starvation [40], TNF-α [41, 42], LPS [43]. Also, TNF-α was reported to induce cell death via a ROS/JNK c-Jun N-terminal kinase)-dependent mechanism [41]. This study showed the suppression of TNF-α by rFm-fortilin (FL). Therefore, it infers that the rFm-fortilin (FL) could protect the cytotoxicity of the HEMA via inhibition of the release of TNF-α, which activates ROS then lead to cell death. However, an animal model is essential to confirm the rFm-fortilin (FL) could protect the cytotoxicity of the HEMA via inhibition of the release of TNF-α.

Moreover, fortilin was known to involve in human cancer [28, 44]. The interaction between TCTP and p53 was found to inhibit Bax transcriptional activation, then result in antiapoptotic activity [45]. The excess of TCTP can degrade p53, effected on unable to activate apoptosis [46, 47]. The rFm-Fortilin (FL) without glutathione S-transferase (GST) also confirmed antiapoptotic activity and the excess of the rFm-Fortilin (FL) at 10 μg/ml did not cause CHO-K1 mutation at 24–72 h. In comparison, only 1 ng/ml was enough to protect pulp cells from the cytotoxicity of HEMA.

The rFm-GST-TCTP protein has been reported to promote mineral deposition via activation alkaline phosphatase activity and expression of Dentin sialophosphoprotein (DSPP), dentin matrix protein 1 (DMP-1), and bone morphogenetic protein-2 (BMP-2) [48]. Besides, the rFm-Fortilin (FL) without glutathione S-transferase (GST)-in this studied was confirmed to promote cell mineralization.

Mast cells and basophils play roles in inflammatory reactions. The cells induced the secretion of proinflammatory molecules, such as histamine, proteases, prostaglandins, cytokines, and chemokines, which lead to allergic inflammation [49]. Histamine releasing factor (HRF) can activate to release IL-4 and IL-3 and then histamine from basophils and mast cells [50, 51]. HRF was also named TCTP and can bind with Igs and HRF-reactive IgE-activated mast cells in vitro [51]. In addition, Ig-interacting HRF peptides were shown to inhibit IgE/HRF-induced mast cell activation and prevented in vivo cutaneous anaphylaxis and airway inflammation. Therefore, Kashiwakura and colleagues proposed HRF as a potential therapeutic target [51]. In the present study, rFm-Fortilin (FL) suppressed histamine upon activation by LPS, which implies that it could be applied therapeutically without activating allergic responses.

## Conclusions

The recombinant protein rFm-Fortilin (FL) used in this study could promote the growth of HDPCs and reduced HDPC death caused by HEMA in a concentration-dependent manner. Mineralization occurred at a low concentration of the rFm-Fortilin (FL). Allergy activation did not happen via histamine or TNF-α, and the rFm-Fortilin (FL) did not result in genomic mutation. Although rFm-Fortilin showed potential for use in dental cement, experiments in an animal model are essential for investigating other potential effects.

## Supporting information

S1 File (DOCX)
