# Supplementary material for: Biological activities of a recombinant fortilin from Fenneropenaeus merguiensis
Source: PLoS One. 2020 Oct 1;15(10):e0239672. doi: 10.1371/journal.pone.0239672 (PMC7529305; doi:10.1371/journal.pone.0239672)
Supplement: S1 File — (DOCX) [file pone.0239672.s001.docx]

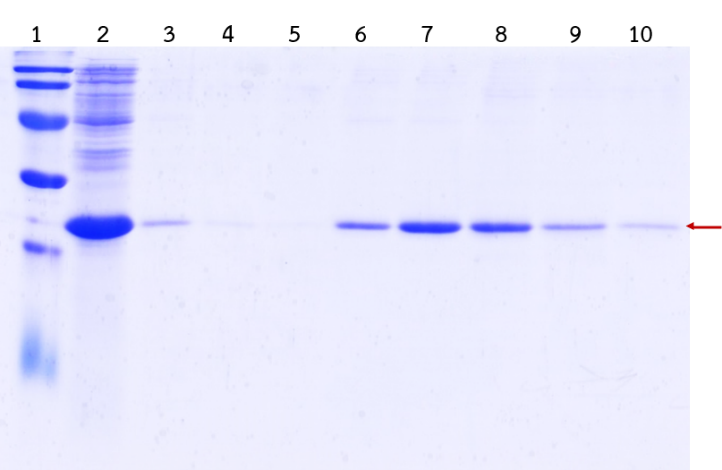


S2A


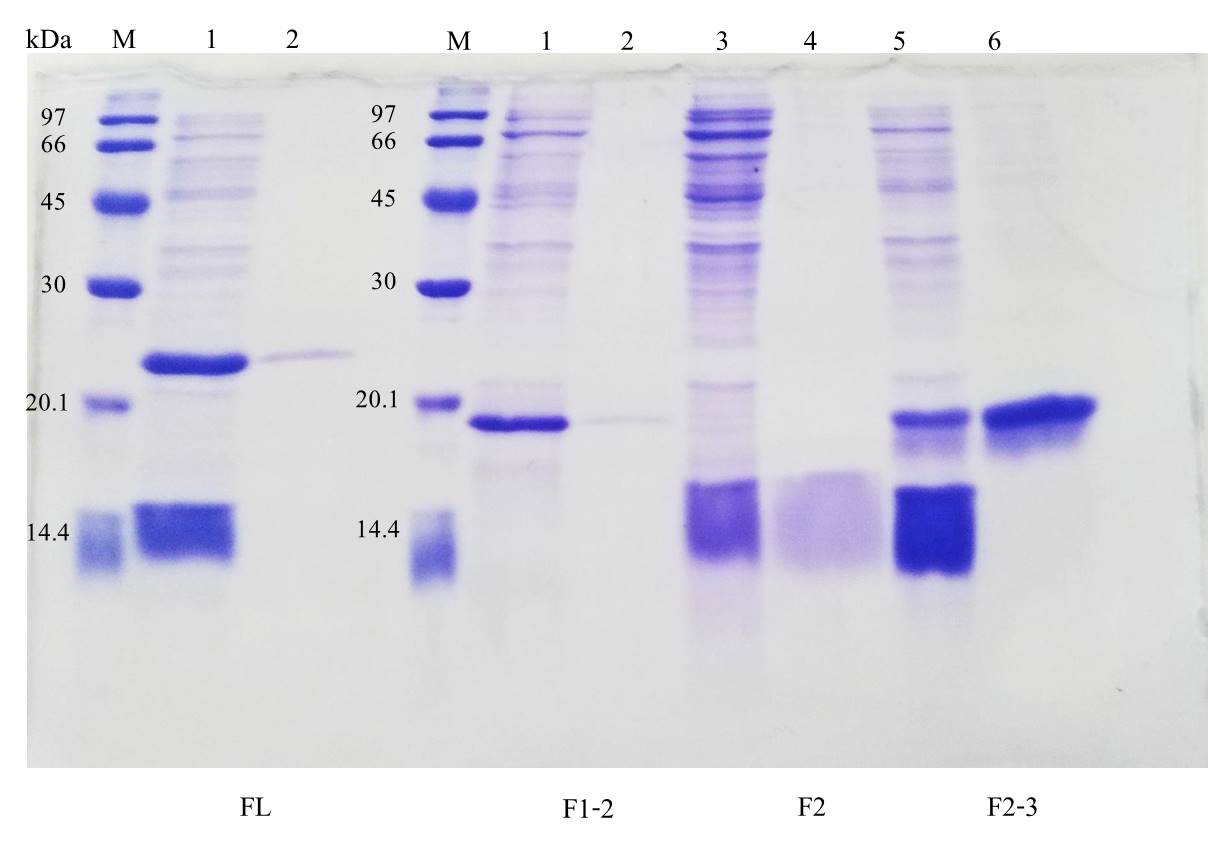


S 2B

**S 2**. **r*Fm*-Fortilin and its fragments separated on 15% SDS-PAGE**

**(A)** rFm-Fortilin (FL) transformed in *E. coli* BL21(DE3) harboring pET29a-Fortilin-Full Length was analyzed on 15% SDS-PAGE and stained with Coomassie brilliant blue. Lane M: Molecular weight marker; Lane 1: Soluble fraction of crude extract; Lane 2-4: Eluted fractions from DEAE column; Lane 5-8: Purified r*Fm*-Fortilin from DEAE cellulose column.

**(B)** rFm-Fortilin (F2), rFm-Fortilin (F12), rFm-Fortilin (F23) transformed in *E. Coli* BL21(DE3) harboring pET29a-Fortilin (F2), Fortilin (F12), and Fortilin (F23) were analyzed on 15% SDS-PAGE. Lane M: Molecular weight marker; Lane 1: crude rFm-Fortilin(FL); Lane 2: purified rFm-Fortilin(F2); Lane 3: crude rFm-Fortilin (F12); Lane 4: purified rFm-Fortilin (F12); Lane 5: crude rFm-Fortilin (F23); and Lane 6: purified rFm-Fortilin (F23)
